# Ripening- and Season-Dependent Variation in Polyphenolic Compounds and the Antioxidant Capacity of Sour Cherry (*Prunus cerasus* L.)

**DOI:** 10.3390/antiox15040462

**Published:** 2026-04-08

**Authors:** Anna Pál, Róbert Nagy, Endre Máthé, Péter Keczkó, Péter Sipos

**Affiliations:** 1 Doctoral School of Food Science, University of Debrecen, 4032 Debrecen, Hungary; 2 Institute of Nutrition Science, Faculty of Agriculture, Food Science and Environmental Management, University of Debrecen, 4032 Debrecen, Hungary; nagy.robert@agr.unideb.hu (R.N.); endre.mathe@agr.unideb.hu (E.M.); 3 Penta Familia Cooperative, 4445 Nagycserkesz, Hungary

**Keywords:** sour cherry, polyphenol, antioxidant, organic, conventional, bioactive compounds

## Abstract

Antioxidants play an essential role in human health by reducing damage caused by free radicals. Total polyphenol, flavonoid, and anthocyanin contents and the antioxidant capacity of two sour cherry cultivars (*Cigánymeggy*, *Oblacsinszka*) grown under conventional and organic production systems were evaluated over two consecutive years at different stages of ripening. Results showed that the concentrations of different antioxidant compounds varied during ripening, but more significant differences were observed between the growing seasons, whereas no significant differences were found between the investigated genotypes or cultivation methods. In 2024, total polyphenol values during the harvest period ranged from 1116.33 to 1874.39 mg GAE/100 g DM, while in 2025 they ranged from 909.81 to 1668.96 mg GAE/100 g DM. Polyphenol profile analysis showed that the main polyphenolic compounds of sour cherries, including cyanidin-3-glucosylrutinoside, cyanidin-3-O-rutinoside, cyanidin-3-glucoside, and cyanidin-3-sophoroside, were detected in both years, indicating that the major anthocyanin components were consistently present despite harvesting year effects.

## 1. Introduction

Plant-origin chemically stable antioxidants can neutralize reactive oxygen and nitrogen species, as well as other free radicals, through electron transfer and hydrogen atom donation. They inhibit or delay the initiation of oxidative chain reactions, thereby reducing oxidative damage in cells and food systems. Even at low concentrations, antioxidants can effectively slow or block oxidation, which is crucial for maintaining the redox balance in biological systems and preserving food quality [1].

In food systems, antioxidants primarily delay lipid peroxidation, thereby preventing the formation of secondary oxidation products. In biological systems, antioxidants are defined as substances that delay, prevent, or eliminate oxidative damage to target biomolecules through direct radical scavenging, enhancement of antioxidant defense mechanisms, or reduction in the formation of reactive oxygen species [1,2]. These mechanisms collectively help preserve the structural and functional integrity of cells, and numerous studies show that regular and increased intake of plant-derived antioxidants has significant health-protective effects and reduces the risk of degenerative diseases [3,4]. Numerous fruits have been proven to be rich in antioxidants, among which sour cherry contains an outstanding amount of polyphenolic compounds [5,6,7,8,9]. However, its consumer popularity lags behind that of frequently studied berry fruits, such as blueberries or blackberries.

Sour cherry (*Prunus cerasus* L.) is a tart-flavored fruit belonging to the *Rosaceae* family, which is mainly cultivated in European countries and Asia but distributed worldwide, particularly in temperate regions [10]. In Europe, the area of sour cherry orchards has been gradually decreasing, as old plantations are not being renewed. In the 2024/25 marketing year, production increased moderately compared to 2023, reaching approximately 715,000 tonnes. Within the European Union, most sour cherry production is used for processing, mainly as frozen fruit and fruit juice concentrate, and for jams and preserves [11]. The chemical profile of sour cherry strongly depends on genotype, ripening stage, cultivation practices, and environmental conditions. These factors determine the sugar–acid balance and content of antioxidant and bioactive compounds, which ultimately affect the overall fruit quality and taste [12]. It has a fleshy, juicy texture and a hard, oval stone at the center. The color of the fruit ranges across the red spectrum, from light red to deep burgundy [12,13,14]. Polyphenols, which are secondary metabolites of plants that have been proven to exert positive health effects, are among the most important bioactive compounds of sour cherry [15].

More than 8000 polyphenolic compounds have been identified [15,16]. These compounds play multiple roles in plant life: they contribute to defense mechanisms, exhibit antimicrobial and insecticidal effects, influence cell division and differentiation, and promote cell wall strengthening and lignin synthesis. These compounds also largely determine the taste, aroma, and color of fruits, vegetables, and flowers [17]. The polyphenolic compounds present in sour cherry have been shown to exert positive health effects. According to some studies, they may also play a role in the management of hypertension and can promote muscle recovery [18,19]. Its consumption may also be promising for the prevention and management of various chronic diseases, such as cardiovascular disease, hypertension, diabetes, and gout, as well as for different inflammatory biomarkers [9,19,20,21,22].

The polyphenolic compounds found in the highest amounts in sour cherry are anthocyanins, which accumulate mainly in the skin. Consequently, the skin largely contributes to the fruit’s taste, tartness, and biological value [22,23,24]. These anthocyanin compounds are responsible for the bright red color of sour cherry and have been shown to possess potent antioxidant activity. Cyanidin-3-O-rutinoside is relatively rare in nature; however, in sour cherry, it is the dominant anthocyanin, accounting for up to 80–90% of the total anthocyanin content [10,25,26,27].

The success of cultivation largely depends on cultivar selection and orchard management. Harvesting sour cherry presents significant technological challenges, especially for fruit intended for fresh consumption. Many sour cherry cultivars are attached to the shoots with relatively short pedicels, which complicates both manual and mechanical harvesting. In red-fruited cultivars, the ripening process is characterized by the loss of green color and the development of red coloration, which is associated with chlorophyll degradation and the accumulation of polyphenolic compounds, including anthocyanins. As ripening progresses, the fruits become increasingly soft and highly juicy, which can lead to mechanical damage and spoilage, particularly under intensive transport and processing conditions. Therefore, precise determination of harvest time is critical for maintaining fruit quality and compositional attributes [23,28]. Furthermore, the proper selection of harvest time is critical because sour cherry is classified as a non-climacteric fruit, which means that ripening does not continue after harvest [14]. Several studies in the literature have reported higher dry matter content in organic fruits; however, contradictory results have also been observed. The higher dry matter content observed in organic cultivation is partly explained by the fact that water-soluble mineral fertilizers used in conventional farming increase water uptake, resulting in higher water content in the plant’s soft tissues and, consequently, the phenomenon of “water swelling” [29,30]. In a 2010 study conducted in Hungary, the polyphenols, chlorogenic acid, neochlorogenic acid, and rutin were analyzed in sour cherry fruits. Although no significant differences were found in chlorogenic acid and rutin content between samples from conventional and organic cultivation, considerable variation was observed across harvest years [28]. According to a later study, organic farming systems can significantly affect the phenolic composition of sour cherry fruits. In a multi-year experiment, samples from four sour cherry cultivars (“*Oblacińska*”, “*Kelleris 16*”, “*Pandy 103*”, and “*Debreceni Bötermo*”) grown in organic and conventional orchards were compared. The results showed that, in certain years, organically grown fruits had significantly higher dry matter and polyphenol content. In the organic samples, the concentration of phenolic acids and flavonoids—including quercetin-3-O-rutinoside (main for organic: 3.4 g/100 g FW and main for conventional: 1.6 g/100 g FW)—was notably higher, while among anthocyanins, cyanidin-3-O-rutinoside was the dominant form. The study also highlighted that cultivar and harvest year are significant influencing factors, indicating that long-term investigations are necessary to understand the differences between organic and conventional cultivation [29].

**Figure 1:**
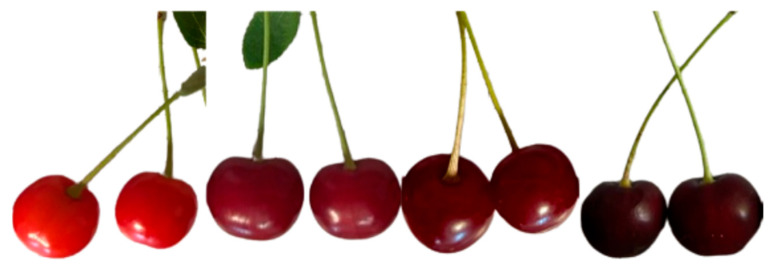
Color changes of sour cherry (*Cigánymeggy*) during ripening. Source: own photo.

The primary aim of the study was to comprehensively evaluate sour cherry fruit at four ripening stages, with particular focus on the compositional characteristics and antioxidant profiles of samples from organic and conventional cultivation (Figure 1). There is limited comprehensive research examining the effects of these cultivation systems on sour cherry growth and fruit quality. The investigations assessed the quality of two cultivars over two consecutive years from seven different orchards. The study focused on measuring total polyphenol, total flavonoid, and total anthocyanin contents, as well as antioxidant capacities (DPPH and FRAP). It was complemented by the polyphenol profiling of four sour cherry cultivars at harvest. Unlike many previous studies conducted on small experimental plots or single factors, this research was conducted on commercial orchards under real agricultural conditions, yielding results directly applicable to practical cultivation and processing. By evaluating multiple factors simultaneously—including ripening state, cultivation system, cultivar, and harvest year—the study offers novel insights into how these variables interact to affect the antioxidant profile of sour cherries. These findings also provide a scientific basis for optimizing harvest timing and fruit selection for functional food production, accounting for seasonal and varietal variability.

We hypothesize that the compositional characteristics of sour cherry—particularly the concentration of bioactive compounds—vary significantly over two consecutive years as ripening progresses, and that these changes manifest differently in samples from organic and conventional cultivation.

To achieve the above research objectives, we addressed the following questions:

- How do the bioactive components (total polyphenol, flavonoid, and anthocyanin contents, as well as DPPH and FRAP values) change during ripening in individual cultivars?
- What differences can be expected for in-total polyphenol, flavonoid, and anthocyanin contents, as well as DPPH and FRAP values of harvested sour cherry due to harvesting year and genotype, and does farming method (organic and conventional cultivation) modify them?
- To what extent do the polyphenol profile component contents of the sour cherry samples (four in total) examined at harvest differ between the two years?

## 2. Materials and Methods

### 2.1. Research Material

The research material consisted of sour cherry (*Prunus cerasus* L.) fruits. Two sour cherry cultivars, *Cigánymeggy* and *Oblacsinszka,* were included in the tests. Their cultivation sites, GPS coordinates, and cultivation methods are presented in Table 1.

**Figure 2:**
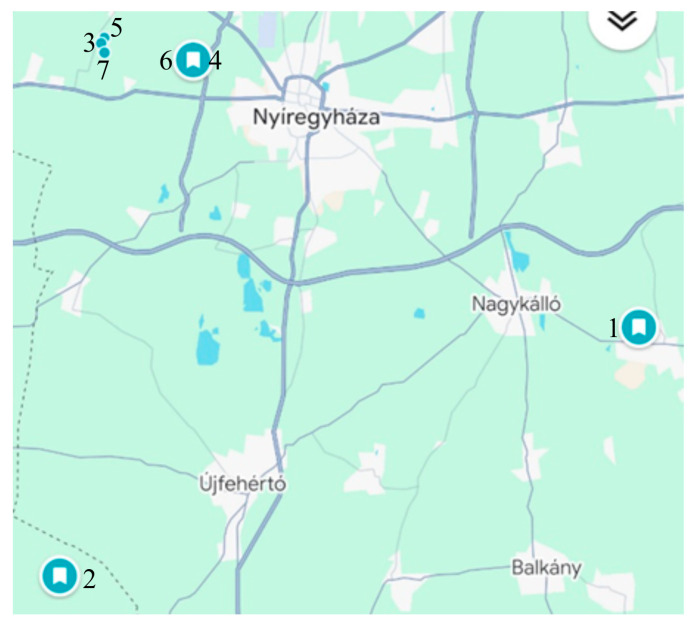
Location of sampling sites (source: Google Maps) (Created in Canva, Pal, A. (2026) https://www.google.com/maps accessed on 25 March 2026) Site identifiers and coordinates are shown in Table 1.

The research materials were sampled from orchards cultivated on sandy soils in the Nyírség region of eastern Hungary (Figure 2). Fruit samples were randomly collected from each tree, a total amount of 1.5–2 kg. During sampling, cherries were not taken from a single tree, but from the same 6–7 pre-selected trees at all sampling times to minimize variability arising from differences between individual trees. Fruits were collected from parts of the tree accessible from the ground, including both areas close to the trunk and those farther away.

Fruits were sampled at different times over the two years to align with the expected harvest period. Sampling was conducted weekly. The first sampling was performed when the sour cherries began to ripen, i.e., upon the appearance of light red coloration.

During the two-year study, the sampling dates were as follows:

- Year (2024)—3 June, 10 June, **17 June**, 24 June
- Year (2025)—11 June, 18 June, **24 June**, 30 June

The dates indicating suitability for mechanical harvesting are highlighted in **bold**.

The sour cherry cultivars examined (“*Cigánymeggy*” and “*Oblacsinszka*”) have different ripening periods; however, sampling was conducted on the same day for each session. As a result, the sour cherries collected at each sampling point were not necessarily at the same physiological maturity stage. Laboratory analyses were initiated immediately after sampling. In total, 56 samples were analyzed over the two ripening periods. All samples were analyzed in triplicate.

Harvesting did not occur on a single day, but over a short, multi-day interval. During data processing, these values were treated uniformly as the harvest time. Given that the investigated sour cherry cultivars have different ripening dynamics, comparisons were made using the exact sampling dates and harvest phases rather than identical physiological maturity stages.

### 2.2. Weather Conditions in the Two Study Years

Due to a mild winter and an early spring, vegetative development of trees in Hungary started approximately three weeks earlier than usual. In mid-March, cold waves affected certain regions of the country, causing frost damage in some orchards and reducing fertility. During flowering, extremely warm and dry weather prevailed, with low relative humidity. Although pollinators were active, the buds dried out rapidly, reducing the chances of successful fertilization and lowering the expected yield. This was followed by a cold, overcast period of approximately four weeks that threatened fruit set. However, subsequent rainfall had a positive effect on fruit size. Overall, the crop for the 2024/25 marketing year is expected to exceed the 2023/24 yield [11] slightly. In the eastern region of Hungary (Nyíregyháza area), the sour cherry growing season (May–June) of 2024 was characterized by warmer-than-average and notably dry weather, with precipitation significantly below the long-term average and a high number of sunshine hours. However, a brief rainfall event also occurred in this region. Prolonged dry periods and high evaporative demand created particularly unfavorable conditions for fruit development [30].

In 2025, sour cherry yield was strongly influenced by weather conditions as spring frosts caused significant damage in the orchards, resulting in a harvest substantially below the 60–70 thousand tons typical of previous favorable years. Frost damage particularly affected early-ripening cultivars, and so harvesting was primarily carried out from mid- and late-ripening varieties. The impact of adverse weather was observed not only in Hungary but also in the central European producing countries, leading to reduced international supply and increased demand for Hungarian sour cherries [31]. In the same region, the 2025 growing season remained precipitation-deficient, with rainfall occurring in a spatially and temporally uneven pattern, typically in the form of short, intense showers. Combined with elevated temperatures, these conditions further increased water stress during the vegetation period [32].

### 2.3. Chemicals

The following chemicals were used in the analyses: Folin–Ciocalteu reagent, gallic acid, sodium carbonate (Na_2_CO_3_), methanol (99.8%), catechin (trans-3,3,4,5,7-pentahydroxyflavan), sodium nitrite (NaNO_2_), aluminum chloride (AlCl_3_), sodium hydroxide (NaOH), hydrochloric acid (HCl 37%), acetic acid, sodium acetate, ferric chloride (FeCl_3_), TPTZ (2,4,6-tripyridyl-s-triazine), ascorbic acid, 2,2-diphenyl-1-picrylhydrazyl (DPPH), Trolox, ethanol. All chemicals used in the study were obtained from VWR International (Radnor, PA, USA) except for the Folin–Ciocalteu reagent, which was purchased from Chem-Lab NV, (Zedelgem, Belgium), and catechin, which was obtained from Sigma-Aldrich (St. Louis, MO, USA). Polyphenol standards were obtained from Merck (Sigma-Aldrich) (Burlington, MA, USA).

### 2.4. Chemical Analysis

#### 2.4.1. Determination of Dry Matter Content

The analysis was performed in a drying oven. The Petri dishes used for the measurements were dried at 105 °C for 30 min, then cooled in a desiccator. The balance was then zeroed, and approximately 5 g of the prepared, homogenized samples was placed into the Petri dishes, also weighed to four decimal places. The samples were then dried in the oven at 105 °C for 4 h, followed by cooling in a desiccator. After cooling, the combined weight of the Petri dish and the sample was measured again, to 4 decimal places. The moisture/dry matter content was then calculated from these measurements and expressed as a percentage [8].

#### 2.4.2. Determination of Total Polyphenol and Flavonoid Contents

The sample preparation for determining total polyphenol and flavonoid contents was identical. After removing the stones, the sour cherry samples were homogenized and used to prepare the solution. For extraction, 5 g of the sample was mixed with 50 mL of an 80:20 methanol–distilled water solution. The samples were extracted for 30 min with intermittent shaking every 5 min, followed by centrifugation for 6 min at 4020 rpm [33]. Total polyphenol content was determined using the Folin–Ciocalteu method, as developed by Singleton and Rossi (1965). A gallic acid stock solution was used to prepare calibration solutions. For reaction mixtures, 0.5 mL of either calibration solution or sample was placed in a 10 mL test tube, followed by the addition of 2.5 mL of 0.2 N Folin reagent. After a 5 min incubation, 2 mL of 75 g/L Na_2_CO_3_ solution was added to each mixture, and the samples were kept in the dark for 2 h. After this period, absorbance was measured at 760 nm using a T60U spectrophotometer (PG Instruments Limited, Lutterworth, UK). Results were expressed as gallic acid equivalents per 100 g of dry matter (GAE/100 g DM) [33].

To determine total flavonoid content, catechin was used as the stock standard. In each test tube, 4 mL of distilled water was added, followed by 1 mL of the sample. Subsequently, 0.3 mL of 5% NaNO_2_ solution was added to the mixtures. After a 5 min incubation, 0.3 mL of 10% AlCl_3_ solution was added to each tube. One minute later, 2 mL of 1 M NaOH solution and 2.4 mL of distilled water were added to each mixture. The absorbance of the samples was measured immediately at 510 nm using a T60U UV -VIS spectrophotometer (PG Instruments, Lutterworth, UK), and expressed as catechin equivalents per 100 g of dry matter (CE/100 g DM) [34].

#### 2.4.3. Determination of Total Anthocyanin Content

Total anthocyanin content was determined using the pH differential method. Two buffer solutions were required for the measurement. Potassium chloride buffer (pH 1.0) was used for one solution and sodium acetate buffer (pH 4.5) for the other. Absorbance was measured at 510 and 700 nm for both buffers. The anthocyanin content was calculated using the following formula:

A = (A_510_ − A_700_) pH_1.0_ − (A_510_ − A_700_) pH_4.5_

Results were expressed as anthocyanin content in mg per 100 g of sample (ACC mg/100 g) [35].

#### 2.4.4. Determination of Antioxidant Capacity by DPPH Method

Antioxidant activity was assessed by measuring the photometric absorbance of the 1,1-diphenyl-2-picrylhydrazyl (DPPH) radical, which has a maximum absorbance at 517 nm. For the assay, 1 g of fresh sour cherry sample was extracted with 40 mL of methanol. The samples were allowed to extract for 30 min with intermittent shaking every 5 min, followed by centrifugation for 6 min at 4020 rpm, and the supernatant was used for the measurement.

A 0.9% methanolic DPPH solution was used to determine antioxidant activity, while a 1 mg/mL methanolic Trolox solution served as the calibration standard. For the measurement, 100 µL of the sample was mixed with 1400 µL of methanol and 1500 µL of DPPH solution. The mixture was incubated in the dark for 30 min, after which absorbance was measured using a spectrophotometer. The results were expressed as mg Trolox per 100 g of dry matter (mg Trolox/100 g DM) [36].

#### 2.4.5. Determination of Antioxidant Capacity by FRAP Method

The ferric reducing ability of plasma (FRAP) method, based on iron-reducing capacity, was developed by Benzie and Strain in 1996. The reduced complex produces a blue color, the intensity of which can be measured spectrophotometrically at 593 nm.

For the assay, 1 g of fresh sour cherry sample was mixed with 40 mL of distilled water. The samples were extracted for 30 min with intermittent shaking every 5 min, followed by centrifugation for 6 min at 4020 rpm; the supernatant was used for measurement. The reaction mixture was prepared in an acetate buffer (pH 3.6), to which FeCl_3_ and TPTZ solutions were added. For calibration, a 1 mg/mL ascorbic acid solution was used.

For measurement, 10 µL of the sample was mixed with 65 µL of distilled water and 2250 µL of FRAP reagent. The mixture was incubated for 8 min, after which absorbance was measured using a spectrophotometer. Values were expressed as mg per 100 g of dry matter (mg AA/100 g DM) [37].

For the spectrophotometric measurements, a T60U UV-VIS spectrophotometer (PG Instruments, Lutterworth, UK) was used.

#### 2.4.6. Polyphenol Profile Analysis

A total of four sour cherry samples were selected for analysis at harvest time. The chosen samples included conventional *Cigánymeggy* from Hajdúdorog, as well as organic *Cigánymeggy*, organic *Oblacsinszka*, and conventional *Oblacsinszka* from Nagycserkesz—Halmosbokor.

Secondary metabolites were separated using a Dionex Ultimate 3000RS (Thermo Fisher Scientific, Germering, Germany) UHPLC system. The analytes were separated on a Thermo Accucore C18 chromatographic column (Thermo Fisher Scientific, Germering, Germany) (100 mm × 2.1 mm, 2.6 μm) maintained at 25 °C (±1 °C). The mobile phases consisted of water (A) and methanol (B), both acidified with 0.1% formic acid. The flow rate was 200 μL/min. The gradient program was as follows: 0–3 min, 95% A; 3–43 min, linear gradient to 0% A; 43–61 min, 0% A; 61–62 min, return to 95% A; 62–70 min, 95% A.

The UHPLC system was coupled to a Thermo Q Exactive Orbitrap hybrid mass spectrometer (Thermo Fisher Scientific, Germering, Germany) with an electrospray ionization (ESI) source. Mass spectrometric data were collected in separate runs using positive (4.0 kV) and negative (3.8 kV) ionization modes. The following parameters were applied: acquisition resolution 70,000 (full scan), fragmentation resolution 35,000; collision energy 30 NCE; m/z range 100–1500.

Raw data were processed using TraceFinder 3.1 (Thermo Fisher Scientific, Germering, Germany). Secondary metabolites were identified by comparison with our previous publications and available databases (Metlin, MassBank of North America, mzCloud). For identification, exact mass, isotopic pattern, characteristic fragment ions, and retention time were considered, and the deviation between measured and calculated monoisotopic mass was always less than 5 ppm [38]. Authentic standards were used for the identification of the main compounds, including neochlorogenic acid (5-O-caffeoylquinic acid), catechin, chlorogenic acid (3-O-caffeoylquinic acid), caffeic acid, epicatechin, p-coumaric acid, quercetin-3-O-sophoroside (baimaside), isoquercitrin (quercetin-3-O-glucoside), rutin (quercetin-3-O-rutinoside), and naringenin (4′,5,7-trihydroxyflavanone).

### 2.5. Statistical Analysis

All measurements were performed in triplicate. Results are presented as mean ± standard deviation (SD). The main effects of cultivation year, cultivar, cultivation method and their interactions on the chemical parameters were analyzed by three-way analysis of variance. The evaluation of changes in concentrations of bioactive compounds and antioxidant activity values of single cultivars at different locations were assessed by one-way analysis of variance (ANOVA) and pairwise comparisons of means were performed using Tukey’s post hoc test afterwards. The means of values from different cultivation methods were compared by *t*-test yearly. Pearson’s correlation analysis was used to analyze the relationships among the evaluated parameters.

### 2.6. Artificial Intelligence

During the preparation of this manuscript, the author(s) used Grammarly (https://app.grammarly.com/ accessed on 13 February 2026) for the purposes of text polishing and grammar correcting. The authors have reviewed and edited the output and take full responsibility for the content of this publication.

## 3. Results and Discussion

### 3.1. Changes in Total Polyphenol, Flavonoid, and Anthocyanin Contents, as Well as DPPH and FRAP Values During Fruit Ripening

From cultivation year, cultivar, and cultivation method, the year had the strongest influence on the examined parameters of sour cherry (Table 2). With the exception of the DPPH values, year effect was significant for all measured parameters (total polyphenols, total flavonoids, total anthocyanins, FRAP), indicating the importance of the cultivation year on the levels of bioactive compounds.

The effect of cultivar was significant only in the FRAP values (F-value = 4.7). The effect of the cultivation method was also moderate; it influenced only the total anthocyanin content significantly (F-value = 4.8).

The examination of interactions revealed further relationships. The year × cultivar interaction had a significant effect (F-value = 6.7) on total polyphenol content, while the cultivar × cultivation method interaction affected anthocyanin content (F-value = 11.5).

#### 3.1.1. Total Polyphenol Content

The total polyphenol content (TPC) of the studied fruit cultivars, expressed on a dry-matter basis, exhibited variable patterns over the two consecutive years (2024–2025) (Table 3). In 2024, the highest TPC values were recorded at the first sampling for all samples. At the second sampling, a slight decrease was observed in most samples, except for two: no statistically significant changes were found in the organic *Cigánymeggy* (Nagycserkesz—Antalbokor) and organic *Oblacsinszka* (Nagycserkesz—Halmosbokor) samples during ripening.

For the other samples, initial TPC values decreased, followed by a slight increase at subsequent sampling points. Similarly, in 2025, two samples exhibited no statistically significant change in TPC during ripening: organic *Cigánymeggy* (Nagycserkesz—Antalbokor) and organic *Oblacsinszka* (Nagycserkesz—Antalbokor). In contrast to the previous year, the highest polyphenol contents were not always observed at the first sampling, and some samples showed a mild increase at later stages. For example, the conventional *Cigánymeggy* (Hajdúdorog) increased from 1571.31 ± 50.09 mg/100 g DM at the first sampling to 2006.51 ± 52.43 mg/100 g DM the following week. Similarly, organic *Oblacsinszka* (Nagycserkesz—Halmosbokor) rose from 1499.35 ± 96.22 mg/100 g DM to 1940.48 ± 191.54 mg/100 g DM.

Greater fluctuations were observed in 2025 compared to 2024. Both of the above-mentioned samples showed a decrease after the increase; however, the conventional *Cigánymeggy* (Hajdúdorog) exhibited a slight rise again at the final sampling. For three samples, namely conventional *Cigánymeggy* (Kállósemjén), conventional *Cigánymeggy* (Hajdúdorog), and organic *Oblacsinszka* (Nagycserkesz—Halmosbokor), the lowest TPC values were recorded at the harvest time when expressed on a dry-matter basis.

A 2012 study from Serbia investigating multiple sour cherry cultivars, including *Oblacsinszka* and *Cigánymeggy*, reported that TPC increased with fruit ripening. In the *Oblacsinszka* cultivar, late ripening stages (stages 5–7) showed significantly higher TPC compared to early stages. Similarly, total antioxidant capacity (TAC) increased during ripening, reaching its maximum at stage 7, showing a strong positive correlation with both total phenol and anthocyanin contents. The concentration of anthocyanins increased markedly with ripening, particularly in the later stages, coinciding with the more intense red coloration of the fruit [39]. In a 2010 study conducted in Hungary, several sour cherry cultivars were monitored throughout fruit ripening, and it was concluded that polyphenol content changed differently among the cultivars. While some cultivars showed an increase in polyphenol content as ripening progressed, others exhibited a decrease or only a moderate change [40].

#### 3.1.2. Total Flavonoid Content

The total flavonoid content (TFC) of the investigated fruit cultivars, expressed on a dry-matter basis, also showed a variable pattern over the two consecutive years (2024–2025) (Table 4). In 2024, on the first sampling date, all samples, with one exception, exhibited the highest flavonoid values. At the second sampling, a slight decrease was observed in all samples. During the third sampling, the flavonoid content of some samples continued to decrease, while in three samples (conventional *Cigánymeggy* (Kállósemjén); conventional *Cigánymeggy* (Hajdúdorog); organic *Cigánymeggy* (Nagycserkesz—Antalbokor)), no significant differences were detected in subsequent samplings. After harvest, an increase in flavonoid content was observed in some locations. For instance, the organic *Oblacsinszka* (Nagycserkesz—Halmosbokor) sample showed 432.60 ± 29.80 mg/100 g at harvest, increasing to 604.39 ± 25.84 mg/100 g at the following sampling.

In 2025, the highest flavonoid values were not always recorded at the first sampling date. Instead, after slight fluctuations, nearly all samples showed the highest flavonoid content at harvest. Following the harvest period, flavonoid levels did not decrease significantly, and some samples showed no statistically lower content after harvest.

#### 3.1.3. Total Anthocyanin Content

The changes in anthocyanin content during ripening, expressed on a dry-matter basis, do not necessarily follow color development; in several cases, the fruit darkened without corresponding higher anthocyanin levels (Table 5).

In the 2024 samples, a marked decrease in anthocyanin content at harvest was observed for the organic *Cigánymeggy* (Nagycserkesz—Halmosbokor) and the organic *Oblacsinszka* (Nagycserkesz—Halmosbokor) samples. Specifically, the organic *Cigánymeggy* (Nagycserkesz—Halmosbokor) sample decreased from 499.29 ± 24.37 mg/100 g before harvest to 154.2 ± 42.96 mg/100 g at harvest. Similarly, the organic *Oblacsinszka* (Nagycserkesz—Halmosbokor) sample decreased from 577.12 ± 41.65 mg/100 g to 200.02 ± 69.96 mg/100 g. After the harvest, a significant increase in anthocyanin content was observed in almost all samples.

In 2025, the pattern of anthocyanin accumulation during ripening differed. At harvest, several samples exhibited their highest anthocyanin content. The conventional *Cigánymeggy* (Kállósemjén) sample showed no statistically significant changes in anthocyanin content during ripening.

#### 3.1.4. DPPH Measurement

Changes in antioxidant capacity during ripening were not uniform across cultivars (Table 6). Considering the DPPH results from 2024, it can be stated that for three samples (conventional *Cigánymeggy* (Kállósemjén); organic *Cigánymeggy* (Nagycserkesz—Halmosbokor; conventional *Oblacsinszka* (Nagycserkesz—Halmosbokor)), there were no statistically significant differences in antioxidant capacity across the sampling dates during ripening. The conventional *Cigánymeggy* (Kállósemjén) sample ranged from 1.19 to 2.01 mg Trolox/100 g. The organic *Cigánymeggy* (Nagycserkesz—Halmosbokor) sample showed similar values, ranging from 1.13 to 1.97 mg Trolox/100 g, while the conventional *Cigánymeggy* (Nagycserkesz—Halmosbokor) sample also did not differ considerably, ranging from 1.55 to 2.10 mg Trolox/100 g. For the other samples, minor fluctuations were observed, but, at harvest, the values fell within a higher range.

For 2025, there were no statistically significant differences between the sampling dates for the first and second samplings. However, during the harvest period, the samples showed the highest values, followed by a decline afterward. The highest DPPH value at harvest was measured in sample 2, the conventional *Cigánymeggy* (Hajdúdorog), with a value of 2.78 ± 0.76 mg Trolox/100 g, followed by the organic *Cigánymeggy* (Nagycserkesz—Halmosbokor), and then the conventional *Cigánymeggy* (Kállósemjén).

#### 3.1.5. FRAP Measurement

Changes in antioxidant capacity during ripening were also not uniform across cultivars (Table 7). Considering the FRAP results of 2024, it can be stated that for the four samples (conventional *Cigánymeggy* (Kállósemjén); organic *Cigánymeggy* (Nagycserkesz—Antalbokor); organic *Cigánymeggy* (Nagycserkesz—Halmosbokor); conventional *Oblacsinszka*—(Nagycserkesz—Halmosbokor)), there were no statistically significant differences in FRAP values across sampling dates during ripening. The conventional *Cigánymeggy* (Kállósemjén) sample ranged from 11.93 to 20.09 mg AA/100 g. The organic *Cigánymeggy* (Nagycserkesz—Halmosbokor) sample showed similar values, ranging from 11.27 to 19.71 mg AA/100 g, while the conventional *Oblacsinszka* (Nagycserkesz—Halmosbokor) sample also did not differ considerably, with values between 15.49 and 20.99 mg AA/100 g. For the other samples, minor fluctuations were observed, but, at harvest, the values fell within a higher range.

For 2025, fluctuations were observed for all samples. There were no statistically significant differences between the first and second sampling dates. However, during the harvest period, the samples showed the highest values, followed by a decrease afterward. The highest FRAP value at harvest was measured in the conventional *Cigánymeggy* (Hajdúdorog) sample, with a value of 27.75 ± 7.57 mg AA/100 g, followed by the organic *Cigánymeggy* (Nagycserkesz—Halmosbokor), and then the conventional *Cigánymeggy* (Kállósemjén).

### 3.2. Comparison of Total Polyphenol, Flavonoid, Anthocyanin, DPPH, and FRAP Contents at Harvest Between 2024 and 2025

The comparison of total polyphenol content between the 2024 and 2025 samples revealed a cultivar-dependent year effect. Statistical analysis showed a significant difference for the conventional *Cigánymeggy* (Kállósemjén) sample (*p* < 0.001), as well as for the organic *Oblacsinszka* (Nagycserkesz—Halmosbokor) (*p* < 0.05) and the conventional *Oblacsinszka* (Nagycserkesz—Halmosbokor) samples (*p* < 0.01). In contrast, for the other samples, no statistically significant differences were observed between the two harvest years.

Regarding total flavonoid content, significant differences were detected between the two years for all samples. Flavonoid values were higher in 2025. The organic *Oblacsinszka* (Nagycserkesz—Antalbokor) sample showed 216.11 ± 34.77 mg CE/100 g in 2024, while in 2025, the harvest value increased to 1074.97 ± 73.66 mg/100 g, representing the largest statistically significant difference between the two years.

For anthocyanin content, no statistically significant differences were observed in any case. The organic *Oblacsinszka* (Nagycserkesz—Halmosbokor) and conventional *Oblacsinszka* (Nagycserkesz—Halmosbokor) samples showed no significant differences at harvest, whereas the other samples exhibited measurable differences.

DPPH was the only parameter for which no statistically significant differences were observed between the two years at the harvest date.

In FRAP measurements, differences between the two years were observed in four of the seven samples. The conventional *Cigánymeggy* (Hajdúdorog) sample had a FRAP value of 11.03 ± 0.23 mg AA/100 g in 2024, which increased to 28.94 ± 2.10 mg AA/100 g in 2025. For the organic *Cigánymeggy* (Nagycserkesz—Halmosbokor) sample, a lower FRAP value of 19.94 ± 0.51 mg AA/100 g was measured in 2024, increasing to 28.77 ± 1.38 mg/100 g at harvest in 2025.

### 3.3. Differences in Total Polyphenol, Flavonoid, and Anthocyanin Contents, as Well as DPPH and FRAP Values at Harvest Between Organic and Conventional Cultivation

In the 2024 harvest, when comparing the antioxidant profiles of organic and conventional samples, we found no statistically significant differences in total polyphenol and flavonoid contents or DPPH values (Table 8). However, differences were detected in anthocyanin content (*p* < 0.05) and FRAP values (*p* < 0.05), indicating that the effect of cultivation method was minimal but measurable. In the 2025 harvest, the antioxidant profiles of organic and conventional samples were generally similar, with only a minimal but statistically significant difference observed in DPPH values (*p* < 0.05). A previous study analyzing organic and conventional sour cherries from 2016 to 2018 reported that total polyphenol content was significantly higher in organically grown fruit. In the same study, flavonoid values were also significantly higher in organic sour cherries (organic: 81.7 ± 8.1 g/100 g FW; conventional: 66.7 ± 6.0 g/100 g FW). Regarding anthocyanin compounds, the organic sour cherries showed significantly higher values (organic: 73.1 ± 7.9 g/100 g FW; conventional: 59.6 ± 6.1 g/100 g FW). It should be noted that the earlier study reported values based on fresh weight, whereas our study presents results on a dry-matter basis, which may explain the differences from our findings [29]. In another study comparing organic and conventional sour cherries, organic sour cherries had significantly higher total sugar and anthocyanin content than conventional sour cherries. At the same time, the conventional fruits showed significantly higher dry matter and organic acid contents. No statistically significant differences were observed between the two cultivation methods in terms of vitamin C, total polyphenol, total phenolic acid, or total flavonoid content [41].

### 3.4. Differences in Total Polyphenol, Flavonoid, and Anthocyanin Contents, as Well as DPPH and FRAP Values at Harvest Between the Two Genotypes

In the 2024 growing season, differences between genotypes were observed in flavonoid content (*p* < 0.05) and FRAP values (*p* < 0.05). In contrast, no statistically significant differences were detected in total polyphenol and anthocyanin contents, or in DPPH values (*p* > 0.05). In the 2025 growing season, none of the investigated antioxidant parameters showed statistically significant differences between genotypes (*p* > 0.05). In a study conducted in Serbia in 2012, different sour cherry cultivars were examined, and the results showed significant differences among genotypes in total phenol and flavonoid contents (*p* < 0.05), whereas for certain antioxidant parameters, no statistically significant differences were detected [39]. In a 2017 study conducted in India, significant differences were observed among the examined genotypes in polyphenol and antioxidant contents as the TPC and TAC values of the fresh fruits varied depending on the genotype, and this trend was maintained during jam preparation [42]. In the 2012 Serbian study, which also examined *Oblacsinska* and *Cigánymeggy*, *Oblacsinska* had the highest polyphenol content (fw), followed by *Cigánymeggy*. The authors emphasized that, as is well known, genetic traits as well as agronomic and environmental factors significantly affect phenolic composition, and, consequently, the nutritional quality of fruits, and their results also support this [39]. In contrast, in our case, there was no significant difference in polyphenol content at harvest.

### 3.5. Pearson’s Correlation Analysis Between the Content of Bioactive Compounds and Antioxidant Activity of Sour Cherries

The correlation analysis of the results of matured sour cherry samples shows significant positive correlation between total flavonoid and total anthocyanin content (r = 0.86, *p* < 0.01) (Table 9). A significant positive relationship was also observed between total flavonoids and FRAP values (r = 0.66, *p* < 0.01), suggesting that flavonoids contribute substantially to the antioxidant capacity of the samples. A moderate, significant positive correlation was found between total anthocyanins and FRAP (r = 0.48, *p* < 0.01), while total flavonoids and DPPH showed a weak but significant correlation (r = 0.36, *p* < 0.05). Total polyphenol content showed non-significant correlations with the other antioxidant parameters, as did the DPPH and FRAP values (r = 0.18, ns), indicating that the relationships among these parameters may not be strictly linear and that other compounds may also influence antioxidant activity.

### 3.6. The Differences in the Polyphenol Profile Components of the Sour Cherries Examined at Harvest (Four Samples in Total), Between the Two Years Evaluated

The analyzed samples included conventional *Cigánymeggy* (Hajdúdorog), organic *Cigánymeggy* (Nagycserkesz—Halmosbokor), organic *Oblacsinszka* (Nagycserkesz—Halmosbokor), and conventional *Oblacsinszka* (Nagycserkesz—Halmosbokor).

According to the literature, the main phenolic compounds in sour cherries include catechins, quercetin-3-glucoside, quercetin-3-rutinoside, and neochlorogenic acid. Among the anthocyanins, the dominant compounds are cyanidin derivatives, such as cyanidin-3-glucosylrutinoside and cyanidin-3-rutinoside, while smaller amounts of cyanidin-3-glucoside, cyanidin-3-sophoroside, and peonidin-3-glucoside are also present [43,44,45]. Cyanidin-3-glucosylrutinoside accounts for a major portion of the cherry’s anthocyanin content, often representing 80–90% [46].

During polyphenol profile analysis, various proanthocyanidins were detected in all four samples examined in both years. In addition, catechin, chlorogenic acid, and neochlorogenic acid were identified in each sample, along with several anthocyanin and flavonol derivatives, including cyanidin-3-O-sophoroside, cyanidin-3-O-(2G-glucosyl)rutinoside, cyanidin-3-O-rutinoside, cyanidin-3-O-(2G-xylosyl)-rutinoside, pelargonidin-3-O-(2G-glucosyl)rutinoside, peonidin-3-O-rutinoside, as well as isoquercitrin (quercetin-3-O-glucoside), rutin (quercetin-3-O-rutinoside), and kaempferol-3-O-rutinoside.

However, in our study, differences in anthocyanin content were observed between samples from the two years, consistent with other studies that reported vintage-dependent variations in the anthocyanin profile of sour cherries [46].

**Figure 3:**
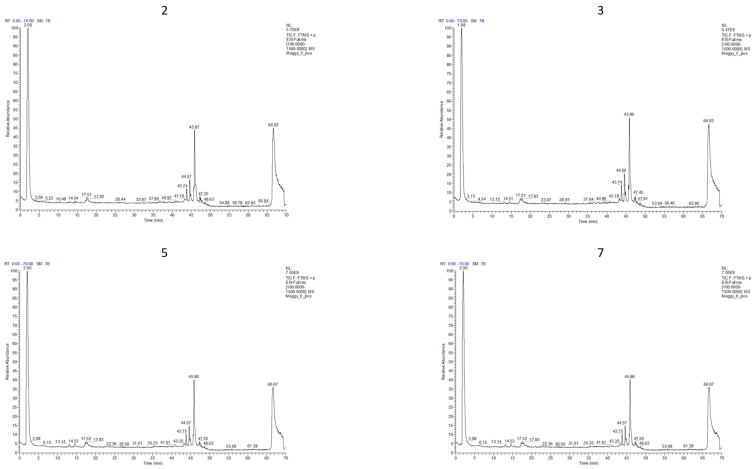
Polyphenol profile values of the four samples (2: conventional *Cigánymeggy* (Hajdúdorog), 3: conventional *Oblacsinszka* (Nagycserkesz—Halmosbokor), 5: organic *Cigánymeggy* (Nagycserkesz—Halmosbokor), 7: organic *Oblacsinszka* (Nagycserkesz—Halmosbokor)) in positive ion mode ([M+H]+) (2024).

Cyanidin-3-O-glucoside (kuromanin), isorhamnetin-3-O-rutinoside-7-O-rhamnoside, as well as certain hydroxycinnamic acid derivatives—namely cis- and trans-4-O-glucosyl-p-hydroxycinnamate and 4-O-glucosyl-p-hydroxydihydrocinnamate—were detected exclusively in the 2024 samples (Figure 3). The occurrence of isorhamnetin-3-O-rutinoside-7-O-rhamnoside was characteristic only for the samples originating from the Halmosbokor site.

**Figure 4:**
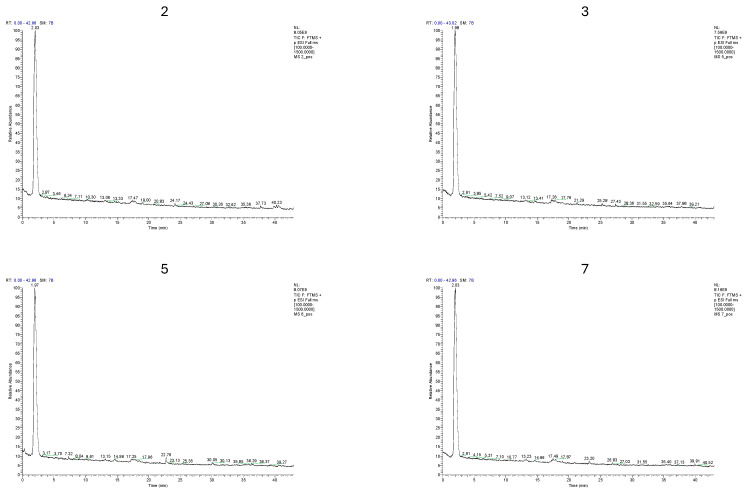
Polyphenol profile values of the four samples (2: conventional *Cigánymeggy* (Hajdúdorog), 3: conventional *Oblacsinszka* (Nagycserkesz—Halmosbokor), 5: organic *Cigánymeggy* (Nagycserkesz—Halmosbokor), 7: organic *Oblacsinszka* (Nagycserkesz—Halmosbokor)) in positive ion mode ([M+H]+) (2025).

Furthermore, the 2025 samples were characterized by the presence of quercetin-3-O-sophoroside (baimaside), naringenin chalcone-O-hexoside, quercetin-O-rutinoside-O-hexoside, as well as p-coumaric acid, 3-O-feruloylquinic acid, and caffeic acid (Figure 4) [47].

## 4. Conclusions

Our results shows that cultivation year has the most significant influence on the concentration of bioactive compounds and the antioxidant activity of sour cherry, while the effects of genotype and cultivation method were only occasionally proved, both in itself or in interaction with other factors. The dynamics of bioactive compounds in the examined sour cherry cultivars did not follow a uniform trend during ripening. In our study, values were presented on a dry-matter basis, whereas most previous studies report data based on fresh weight. Sampling was performed randomly from hand-accessible parts of the trees, meaning that fruits of identical color were not always selected. Consequently, our results reflect the average bioactive profile of the entire fruit population.

Analysis of anthocyanins showed that random sampling does not necessarily reproduce the consistently increasing trends often reported in the literature. It is generally observed that anthocyanin content increases as fruit color darkens; however, since fruits on the tree are not always at the same stage of ripeness, our results may differ from the literature values.

Comparing years revealed that harvesting year significantly affects the measured parameters. While total polyphenol content did not show significant differences between the two study years, total flavonoid content was significantly higher in all samples in 2025.

A comparison of the antioxidant profiles of organic and conventionally grown cherries showed that cultivation method had no significant effect on the parameters examined. In 2024, total polyphenol and flavonoid content, as well as DPPH values, did not differ significantly between the two cultivation methods, whereas minor but statistically significant differences were observed for anthocyanin content and FRAP values. In 2025, the antioxidant profiles of organic and conventional samples were virtually identical, with only minimal differences in DPPH values.

During the analysis of the polyphenol profile, we observed that the major polyphenolic compounds characteristic of sour cherries were present in all examined years. Minor differences were noted between years; however, these did not involve the predominant polyphenols, meaning that the main components were consistently detectable in both years.

Analysis of the genotypes indicated that most antioxidant parameters did not differ significantly between the *Cigánymeggy* and *Oblacsinszka* cultivars at harvest. In 2024, differences were observed for flavonoid content and FRAP values, but in 2025, there were no statistically significant differences. It is important to note that these results do not exclude the influence of genotype; however, in the examined samples, harvest year, environmental, and agrotechnical factors likely had a stronger impact on the antioxidant profile.

Both the sampling strategy and the extraction procedure can significantly influence measured concentrations of bioactive compounds; therefore, it is recommended that future studies use a standardized, or at least well-defined, representative sampling and extraction protocol. Future research should evaluate bioactive compound dynamics in greater detail, considering the combined effects of cultivation method, genotype, resistance, and tolerance, and use standardized sampling protocols to obtain a more accurate picture of changes during ripening.

Overall, our examinations demonstrate that the year is the factor that has the strongest influence on the amount of bioactive compounds in sour cherry, and the cultivation method had only a negligible effect. The dynamics of bioactive compound synthesis in sour cherry cultivars during ripening do not follow a uniform pattern, and the sampling strategy have significant impact on the observed values. Our study presents the changes in bioactive compounds of sour cherries grown under real, large-scale production conditions during ripening, which—in contrast to the results experienced in small-plot, set-up experimental plots—show the expected changes in real vegetations. Therefore, our results can contribute to the optimization of harvest timing. Further research is needed to obtain a more precise understanding of the changes in bioactive compounds in sour cherries, with careful attention to standardized sampling and consideration of environmental and agrotechnical factors.

## Figures and Tables

**Table 1:** Cultivation sites, GPS coordinates, and cultivation methods of sour cherry cultivar.

No.	Cultivar	Cultivation Site	GPS Coordinates	Cultivation Method
1	*Cigánymeggy*	Kállósemjén	(47.865666, 21.918525)	Conventional
2	*Cigánymeggy*	Hajdúdorog	(47.7663954, 21.5699698)	Conventional
3	*Oblacsinszka*	Nagycserkesz—Halmosbokor	(47.979033, 21.594895)	Conventional
4	*Cigánymeggy*	Nagycserkesz—Antalbokor	(47.9723725, 21.6502661)	Organic
5	*Cigánymeggy*	Nagycserkesz—Halmosbokor	(47.9811581, 21.5967899)	Organic
6	*Oblacsinszka*	Nagycserkesz—Antalbokor	(47.9723725, 21.6502661)	Organic
7	*Oblacsinszka*	Nagycserkesz—Halmosbokor	(47.9752898, 21.5969143)	Organic

**Table 2:** Effects of year, cultivar, and cultivation method on polyphenol, flavonoid, anthocyanin content, and antioxidant activity (DPPH, FRAP) in sour cherry.

Indicator	Main Effects			Interaction Effects			
	Y	C	CM	Y:C	Y:CM	C:CM	Y:C:CM
Total polyphenol content	13.2 ***	0.1 ^ns^	0.8 ^ns^	6.7 **	0.2 ^ns^	5.0 ^ns^	0.4 ^ns^
Total flavonoid content	156.5 ***	0.6 ^ns^	0.6 ^ns^	1.5 ^ns^	0.0 ^ns^	1.4 ^ns^	0.1 ^ns^
Total anthocyanin content	49.9 ***	0.9 ^ns^	4.8 *	2.7 ^ns^	3.0 ^ns^	11.5 **	0.3 ^ns^
DPPH	2.8 ^ns^	2.6 ^ns^	2.0 ^ns^	0.9 ^ns^	2.9 ^ns^	0.8 ^ns^	0.0 ^ns^
FRAP	33.8 ***	4.7 *	0.8 ^ns^	1.6 ^ns^	6.7 ^ns^	0.0 ^ns^	0.0 ^ns^

Y: year, C: cultivar, CM: cultivation method ***: *p*-value < 0.001, **: *p*-value < 0.01, *: *p*-value < 0.05, ns: not statically significant.

**Table 3:** Total polyphenol content (mg GAE/100 g DM) during ripening in 2024/2025.

Total Polyphenol Content (mg GAE/100 g DM) During Ripening in (2024/2025)							
No.	Cultivation method	Cultivar	Cultivation site	3 June 2024	10 June 2024	17 June 2024	24 June 2024
1	Conventional	*Cigánymeggy*	Kállósemjén	1268 ± 115 ^a^	950 ± 101 ^b^	1210 ± 45 ^ab^	1191 ± 181 ^ab^
2			Hajdúdorog	1376 ± 74 ^a^	1047 ± 106 ^b^	1116 ± 53 ^b^	769 ± 84 ^c^
3		*Oblacsinszka*	Nagycserkesz—Halmosbokor	1744 ± 110 ^a^	1257 ± 125 ^b^	1651 ± 119 ^a^	1764 ± 123 ^a^
4	Organic	*Cigánymeggy*	Nagycserkesz—Antalbokor	1351 ± 46 ^a^	1017 ± 60 ^a^	1197 ± 339 ^a^	944 ± 31 ^a^
5			Nagycserkesz—Halmosbokor	1642 ±78 ^a^	913 ± 94 ^b^	1566 ± 366 ^a^	1390 ± 156 ^ab^
6		*Oblacsinszka*	Nagycserkesz—Antalbokor	1624± 149 ^a^	968 ± 264 ^b^	1174 ± 48 ^ab^	1173 ± 178 ^ab^
7			Nagycserkesz—Halmosbokor	1539 ± 65 ^a^	1126 ± 151 ^a^	1874 ± 632 ^a^	1713 ± 117 ^a^
No.	Cultivation method	Cultivar	Cultivation site	11 June 2025	18 June 2025	24 June 2025	30 June 2025
1	Conventional	*Cigánymeggy*	Kállósemjén	1424 ± 104 ^ab^	1485 ± 262 ^a^	910 ± 32 ^c^***	1060 ± 11 ^bc^
2			Hajdúdorog	1571 ± 50 ^b^	2007 ± 52 ^a^	941 ± 158 ^d^	1298 ± 68 ^c^
3		*Oblacsinszka*	Nagycserkesz—Halmosbokor	1448 ± 131 ^a^	1428 ± 68 ^a^	994 ± 60 ^b^**	1283 ± 41 ^a^
4	Organic	*Cigánymeggy*	Nagycserkesz—Antalbokor	1398 ± 107 ^a^	1452 ± 73 ^a^	1118 ± 348 ^a^	1024 ± 134 ^a^
5			Nagycserkesz—Halmosbokor	1249 ± 146 ^a^	1578 ± 63 ^a^	1669 ± 521 ^a^	1414 ± 77 ^a^
6		*Oblacsinszka*	Nagycserkesz—Antalbokor	1429 ± 502 ^a^	1597 ± 155 ^a^	1014 ± 102 ^a^	1328 ± 21 ^a^
7			Nagycserkesz—Halmosbokor	1499 ± 96 ^b^	1940 ± 192 ^a^	65 ± 123 ^c^*	1371 ± 75 ^b^

Mean values in the same row with different letters are statistically different from each other. Asterisks (*) indicate the significant differences in polyphenol content between the 2024 and 2025 samples (* *p* < 0.05; ** *p* < 0.01; *** *p* < 0.001).

**Table 4:** Total flavonoid content (mg CE/100 g DM) during ripening in 2024/2025.

Total Flavonoid Content (mg CE/100 g DM) During Ripening (2024/2025)							
No.	Cultivation method	Cultivar	Cultivation site	3 June 2024	10 June 2024	17 June 2024	24 June 2024
1	Conventional	*Cigánymeggy*	Kállósemjén	428 ± 28 ^a^	273 ± 25 ^b^	278 ± 8 ^b^	333 ± 36 ^b^
2			Hajdúdorog	511 ± 3 ^a^	283 ± 18 ^b^	266 ± 29 ^b^	251 ± 14 ^b^
3		*Oblacsinszka*	Nagycserkesz—Halmosbokor	565 ± 15 ^a^	480 ± 17 ^b^	413 ± 43 ^c^	536 ± 7 ^ab^
4	Organic	*Cigánymeggy*	Nagycserkesz—Antalbokor	470 ± 29 ^a^	220 ± 75 ^b^	232 ± 10 ^b^	277 ± 15 ^b^
5			Nagycserkesz—Halmosbokor	565 ±34 ^a^	396 ± 31 ^b^	300 ± 19 ^c^	399 ± 37 ^b^
6		*Oblacsinszka*	Nagycserkesz—Antalbokor	587 ± 35 ^a^	312 ± 13 ^b^	216 ± 35 ^c^	332 ± 43 ^b^
7			Nagycserkesz—Halmosbokor	511 ± 27 ^b^	512 ± 5 ^b^	433 ± 30 ^c^	604 ± 26 ^a^
No.	Cultivation method	Cultivar	Cultivation site	11 June 2025	18 June 2025	24 June 2025	30 June 2025
1	Conventional	*Cigánymeggy*	Kállósemjén	451 ± 4 ^b^	440 ± 64 ^b^	895 ± 69 ^a^***	787 ± 67 ^a^
2			Hajdúdorog	472 ± 32 ^d^	623 ± 14 ^c^	971 ± 35 ^a^***	855 ± 32 ^b^
3		*Oblacsinszka*	Nagycserkesz—Halmosbokor	49 ± 29 ^c^	362 ± 13 ^d^	986 ± 53 ^a^***	755 ± 44 ^b^
4	Organic	*Cigánymeggy*	Nagycserkesz—Antalbokor	419 ± 19 ^b^	352 ± 29 ^b^	722 ± 2 ^a^***	603 ± 117 ^a^
5			Nagycserkesz—Halmosbokor	410 ± 23 ^c^	419 ± 26 ^c^	1235 ±131 ^a^***	991 ± 90 ^b^
6		*Oblacsinszka*	Nagycserkesz—Antalbokor	609 ± 39 ^b^	451 ± 23 ^c^	1075 ± 74 ^a^***	956 ± 39 ^a^
7			Nagycserkesz—Halmosbokor	448 ± 23 ^c^	536 ± 21 ^bc^	678 ± 104 ^ab^*	800 ± 56 ^a^

Mean values in the same row with different letters are statistically different from each other. Asterisks (*) indicate the significant differences in flavonoid content between the 2024 and 2025 samples (* *p* < 0.05; *** *p* < 0.001).

**Table 5:** Total anthocyanin content (mg ACC/100 g DM) during ripening in 2024/2025.

Total Anthocyanin Content (mg ACC/100 g DM) During Ripening (2024/2025)							
No.	Cultivation method	Cultivar	Cultivation site	3 June 2024	10 June 2024	17 June 2024	24 June 2024
1	Conventional	*Cigánymeggy*	Kállósemjén	243 ± 24 ^b^	325 ± 4 ^ab^	219 ± 18 ^b^	421 ± 48 ^a^
2			Hajdúdorog	298 ± 22 ^ab^	420 ± 16 ^a^	267 ± 86 ^ab^	164 ± 9 ^b^
3		*Oblacsinszka*	Nagycserkesz—Halmosbokor	450 ± 75 ^a^	558 ± 19 ^a^	450 ± 59 ^a^	599 ± 29 ^a^
4	Organic	*Cigánymeggy*	Nagycserkesz—Antalbokor	312 ± 40 ^ab^	350 ± 28 ^ab^	246 ± 21 ^b^	369 ± 5 ^a^
5			Nagycserkesz—Halmosbokor	381 ± 32 ^b^	499 ± 24 ^ab^	154 ± 43 ^c^	527 ± 17 ^a^
6		*Oblacsinszka*	Nagycserkesz—Antalbokor	185 ± 15 ^b^	178 ± 40 ^b^	183 ± 32 ^b^	419 ± 27 ^a^
7			Nagycserkesz—Halmosbokor	396 ± 3 ^b^	577 ± 42 ^a^	200 ± 70 ^c^	726 ± 5 ^a^
No.	Cultivation method	Cultivar	Cultivation site	11 June 2025	18 June 2025	24 June 2025	30 June 2025
1	Conventional	*Cigánymeggy*	Kállósemjén	262 ± 3 ^a^	415 ± 67 ^a^	428 ± 27 ^a^*	409 ± 44 ^a^
2			Hajdúdorog	422 ± 28 ^b^	659 ± 77 ^a^	548 ± 27 ^ab^*	486 ± 49 ^ab^
3		*Oblacsinszka*	Nagycserkesz—Halmosbokor	245 ± 11 ^c^	400 ± 8 ^bc^	615 ± 71 ^a^	498 ± 25 ^ab^
4	Organic	*Cigánymeggy*	Nagycserkesz—Antalbokor	230 ± 26 ^b^	432 ± 4 ^a^	452 ± 2 ^a^**	267 ± 19 ^b^
5			Nagycserkesz—Halmosbokor	346 ± 25 ^c^	653 ±16 ^ab^	796 ± 122 ^a^	509 ± 1 ^bc^
6		*Oblacsinszka*	Nagycserkesz—Antalbokor	229 ± 5 ^b^	323 ± 26 ^ab^	474 ± 18 ^a^*	378 ± 75 ^ab^
7			Nagycserkesz—Halmosbokor	311 ± 39 ^c^	678 ± 4 ^a^	415 ± 20 ^b^	408 ± 1 ^b^

Mean values in the same row with different letters are statistically different from each other. Asterisks (*) indicate the significant differences in polyphenol content between the 2024 and 2025 samples (* *p* < 0.05; ** *p* < 0.01).

**Table 6:** DPPH assay (mg Trolox/100 g DM) during ripening in 2024/2025.

DPPH Assay (mg Trolox/100 g DM) During Ripening (2024/2025)							
No.	Cultivation method	Cultivar	Cultivation site	3 June 2024	10 June 2024	17 June 2024	24 June 2024
1	Conventional	*Cigánymeggy*	Kállósemjén	1.99 ± 0.16 ^a^	1.23 ± 0.12 ^a^	2.01 ± 0.64 ^a^	1.19 ± 0.31 ^a^
2			Hajdúdorog	2.14 ± 0.53 ^a^	1.36 ± 0.02 ^b^	1.60 ± 0.13 ^ab^	1.01 ± 0.22 ^b^
3		*Oblacsinszka*	Nagycserkesz—Halmosbokor	1.89 ± 0.8 ^a^	2.10 ± 0.47 ^a^	1.54 ± 0.34 ^a^	1.72 ± 0.05 ^a^
4	Organic	*Cigánymeggy*	Nagycserkesz—Antalbokor	2.16 ± 0.34 ^a^	1.00 ± 0.20 ^c^	1.65 ± 0.10 ^ab^	1.31 ± 0.05 ^bc^
5			Nagycserkesz—Halmosbokor	1.97 ± 0.07 ^a^	1.13 ± 0.52 ^a^	1.73 ± 0.29 ^a^	1.60 ± 0.29 ^a^
6		*Oblacsinszka*	Nagycserkesz—Antalbokor	2.23 ± 0.19 ^a^	1.37 ± 0.32 ^b^	1.98 ± 0.27 ^ab^	1.49 ± 0.37 ^ab^
7			Nagycserkesz—Halmosbokor	1.74 ± 0.18 ^b^	1.79 ± 0.09 ^b^	1.53 ± 0.28 ^b^	2.39 ± 0.10 ^a^
No.	Cultivation method	Cultivar	Cultivation site	11 June 2025	18 June 2025	24 June 2025	30 June 2025
1	Conventional	*Cigánymeggy*	Kállósemjén	0.95 ± 0.33 ^b^	0.92 ± 0.07 ^b^	2.11 ± 0.19 ^a^	0.20 ± 0.10 ^c^
2			Hajdúdorog	1.21 ± 0.26 ^b^	1.35 ± 0.26 ^b^	2.78 ± 0.76 ^a^	0.30 ± 0.02 ^b^
3		*Oblacsinszka*	Nagycserkesz—Halmosbokor	1.54 ± 0.68 ^ab^	1.20 ± 0.67 ^ab^	1.95 ± 0.34 ^a^	0.36 ± 0.15 ^b^
4	Organic	*Cigánymeggy*	Nagycserkesz—Antalbokor	1.09 ± 0.31 ^a^	1.04 ± 0.09 ^a^	1.27 ± 0.34 ^a^	0.20 ± 0.07 ^b^
5			Nagycserkesz—Halmosbokor	1.03 ± 0.26 ^b^	1.28 ± 0.42 ^b^	2.45 ± 0.39 ^a^	0.44 ± 0.13 ^b^
6		*Oblacsinszka*	Nagycserkesz—Antalbokor	1.38 ± 0.29 ^ab^	1.19 ± 0.23 ^b^	1.86 ± 0.05 ^a^	0.23 ± 0.02 ^c^
7			Nagycserkesz—Halmosbokor	0.96 ± 0.21 ^ab^	1.04 ± 0.49 ^ab^	1.28 ± 0.27 ^a^	0.26 ± 0.15 ^b^

Mean values in the same row with different letters are statistically different from each other.

**Table 7:** FRAP assay (mg AA/100 g DM) during ripening (2024/2025).

FRAP Assay (mg AA/100 g DM) During Ripening (2024/2025)							
No.	Cultivation method	Cultivar	Cultivation site	3 June 2024	10 June 2024	17 June 2024	24 June 2024
1	Conventional	*Cigánymeggy*	Kállósemjén	34.9 ± 9.3 ^a^	24.3 ± 16.7 ^a^	17.9 ± 3.8 ^a^	30.2 ± 2.6 ^a^
2			Hajdúdorog	47.3 ± 9.6 ^a^	25.2 ± 2.3 ^b^	11.0 ± 0.2 ^c^	24.8 ± 2.8 ^b^
3		*Oblacsinszka*	Nagycserkesz—Halmosbokor	32.6 ± 2.8 ^a^	26.3 ± 6.8 ^ab^	19.8 ± 5.4 ^b^	30.1 ± 3.1 ^ab^
4	Organic	*Cigánymeggy*	Nagycserkesz—Antalbokor	34.3 ± 0.6 ^a^	19.2 ± 6.1 ^a^	19.7 ± 13.0 ^a^	23.0 ± 2.8 ^a^
5			Nagycserkesz—Halmosbokor	35.6 ± 3.8 ^a^	17.3 ± 9.8 ^c^	19.9 ± 0.5 ^bc^	33.2 ± 5.5 ^ab^
6		*Oblacsinszka*	Nagycserkesz—Antalbokor	61.8 ± 34.7 ^a^	31.9 ± 19.2 ^a^	21.4 ± 5.6 ^a^	35.1 ± 4.6 ^a^
7			Nagycserkesz—Halmosbokor	35.5 ± 8.1 ^a^	26.6 ± 4.7 ^a^	28.8 ± 1.6 ^a^	33.4 ± 4.7 ^a^
No.	Cultivation method	Cultivar	Cultivation site	11 June 2025	18 June 2025	24 June 2025	30 June 2025
1	Conventional	*Cigánymeggy*	Kállósemjén	7.32 ± 2.22 ^d^	23.3 ± 2.7 ^c^	29.2 ± 2.00 ^b^*	40.6 ± 0.3 ^a^
2			Hajdúdorog	6.46 ± 2.62 ^c^	21.8 ± 2.9 ^b^	28.9 ± 2.1 ^a^***	29.5 ± 2.8 ^a^
3		*Oblacsinszka*	Nagycserkesz—Halmosbokor	8.08 ± 3.03 ^b^	25.9 ± 2.8 ^a^	30.8 ± 1.1 ^a^*	29.2 ± 2.7 ^a^
4	Organic	*Cigánymeggy*	Nagycserkesz—Antalbokor	6.01 ± 2.06 ^c^	21.8 ± 2.3 ^b^	24.9 ± 3.5 ^b^	34.7 ± 1.4 ^a^
5			Nagycserkesz—Halmosbokor	8.26 ± 3.97 ^c^	30.4 ± 3.3 ^b^	28.8 ± 1.4 ^b^***	42.2 ± 3.1 ^a^
6		*Oblacsinszka*	Nagycserkesz—Antalbokor	6.29 ± 3.28 ^b^	29.5 ± 3.6 ^a^	29.5 ± 2.8 ^a^	35.8 ± 3.5 ^a^
7			Nagycserkesz—Halmosbokor	6.57 ± 4.53 ^c^	30.5 ± 4.4 ^ab^	26.4 ± 1.9 ^b^	37.8 ± 5.5 ^a^

Mean values in the same row with different letters are statistically different from each other. Asterisks (*) indicate the significant differences in FRAP measurements between the 2024 and 2025 samples (* *p* < 0.05; *** *p* < 0.001).

**Table 8:** Differences between organic and conventional sour cherries in bioactive compounds and antioxidant capacity (2024/2025).

Year	Measurements	Mean for Organic	Mean for Conventional	*p*-Values
2024	Total polyphenol (mg GAE/100 g)	1452.8 ± 291.4	1325.9 ± 72.0	0.47
	Total flavonoid (mg CE/100 g)	295.4 ± 24.1	319.1 ± 26.7	0.53
	Total anthocyanin (mg ACC/100 g)	195.9 ± 44.13	312.1 ± 54.2	0.03 *
	DPPH (mg Trolox/100 g)	17.2 ±2.64	17.2 ± 3.7	0.99
	FRAP (mg AA/100 g)	22.5 ± 4.8	16.3 ± 3.2	0.04 *
2025	Total polyphenol (mg GAE/100 g)	1112.6 ± 135.6	948.1 ± 273.4	0.32
	Total flavonoid (mg CE/100 g)	927.5 ± 45.4	950.6 ± 77.8	0.79
	Total anthocyanin (mg ACC/100 g)	534.3 ± 42.2	530.4 ± 40.4	0.96
	DPPH (mg Trolox/100 g)	17.2 ± 3.9	22.8 ± 2.7	0.04 *
	FRAP (mg AA/100 g)	27.4 ± 2.5	29.6 ± 2.4	0.05

Asterisks indicate statistically significant differences between organic and conventional sour cherries based on *t*-test: * *p* < 0.05.

**Table 9:** Correlations between bioactive compounds and antioxidant capacity in sour cherries (2024/2025).

	Total Polyphenol	Total Flavonoid	Total Anthocyanin	DPPH	FRAP
Total polyphenol	1	−0.17	−0.23	0.03	−0.09
Total flavonoid		1	0.86 **	0.36 *	0.66 **
Total anthocyanin			1	0.36	0.48 **
DPPH				1	0.18
FRAP					1

** Correlation is significant at the 0.01 level (two-tailed). * Correlation is significant at the 0.05 level (two-tailed).

## Data Availability

The original contributions presented in this study are included in the article. Further inquiries can be directed to the corresponding author.

## Abbreviations

The following abbreviations are used in this manuscript:

DPPH	2,2-diphenyl-1-picrylhydrazyl
FRAP	Ferric Reducing Ability of Plasma
GPS	Global Positioning System
DM	Dry matter
TPTZ	2,4,6-tripyridyl-s-triazine
UHPLC	Ultra-High Performance Liquid Chromatography
ESI	Electrospray ionization
NCE	Normalized Collision Energy
SD	Standard Deviation
TPC	Total Polyphenol Content
TFC	Total Antioxidant Capacity
GAE	Gallic Acid Equivalent
AA	Ascorbic Acid equivalents
